# Mechanism of Catalytic Water Oxidation by the Ruthenium Blue Dimer Catalyst: Comparative Study in D_2_O *versus* H_2_O

**DOI:** 10.3390/ma6020392

**Published:** 2013-01-30

**Authors:** Dooshaye Moonshiram, Vatsal Purohit, Javier J. Concepcion, Thomas J. Meyer, Yulia Pushkar

**Affiliations:** 1 Department of Physics, Purdue University, 525 Northwestern West Lafayette, IN 47907, USA; E-Mails: dmoonshi@purdue.edu (D.M.); vatsal.purohit15@gmail.com (V.P.); 2 Department of Chemistry, University of North Carolina at Chapel Hill, Chapel Hill, NC 27599, USA; E-Mails: jconcepc@email.unc.edu (J.J.C.); tjmeyer@unc.edu (T.J.M.)

**Keywords:** Ru complexes, catalysis of water oxidation, deuterium isotope effect, time-resolved X-band EPR spectroscopy, time-resolved resonance Raman, UV-Vis stopped flow kinetics

## Abstract

Water oxidation is critically important for the development of energy solutions based on the concept of artificial photosynthesis. In order to gain deeper insight into the mechanism of water oxidation, the catalytic cycle for the first designed water oxidation catalyst, *cis*,*cis*-[(bpy)_2_(H_2_O)Ru^III^ORu^III^(OH_2_)(bpy)_2_]^4+^ (bpy is 2,2-bipyridine) known as the blue dimer (BD), is monitored in D_2_O by combined application of stopped flow UV-Vis, electron paramagnetic resonance (EPR) and resonance Raman spectroscopy on freeze quenched samples. The results of these studies show that the rate of formation of BD[4,5] by Ce(IV) oxidation of BD[3,4] (numbers in square bracket denote oxidation states of the ruthenium (Ru) centers) in 0.1 M HNO_3_, as well as further oxidation of BD[4,5] are slower in D_2_O by 2.1–2.5. Ce(IV) oxidation of BD[4,5] and reaction with H_2_O result in formation of an intermediate, BD[3,4]′, which builds up in reaction mixtures on the minute time scale. Combined results under the conditions of these experiments at pH 1 indicate that oxidation of BD[3,4]′ is a rate limiting step in water oxidation with the BD catalyst.

## 1. Introduction

Photosynthetic water oxidation is a fundamental process in the biosphere, which results in the sunlight driven formation of O_2_ from water. This process occurs in Photosystem II and has remained unchanged for 2.4 billion years. It created the current oxygen-rich atmosphere of 21% abundance [1]. Mimicking this reaction in a working man-made device would allow for sunlight-to-chemical energy conversion with water providing electrons and protons for the formation of oxygen and reduced chemicals. Such processes are best suited for sustainable and clean generation of H_2_ [2,3,4]. In addition, the byproduct of water oxidation is non-polluting O_2_, which is converted back to H_2_O by respiration and combustion. These processes, however, require efficient, robust and economically feasible catalysts. 

**Figure 1:**
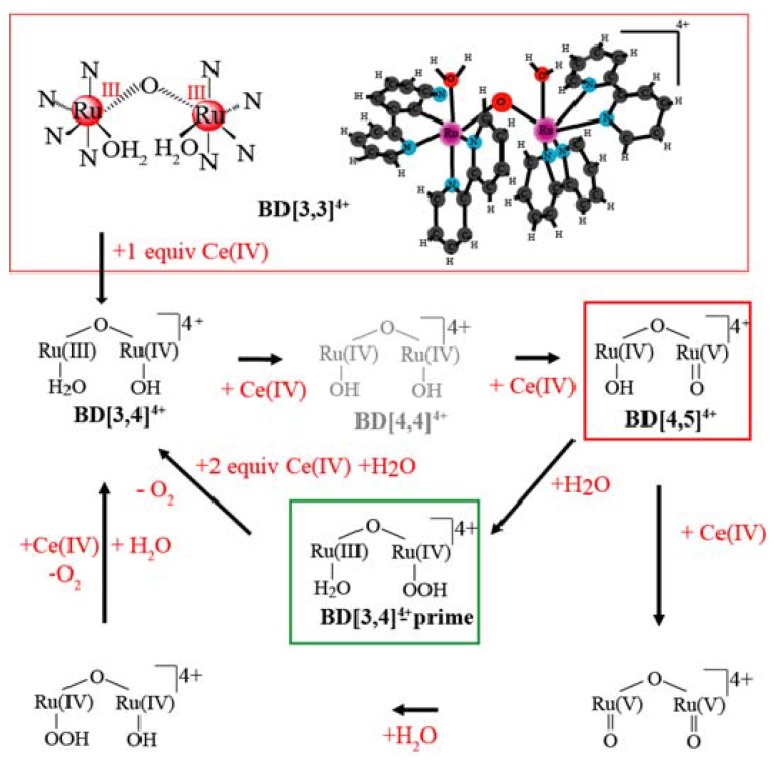
Mechanism of blue dimer (BD) water oxidation.

About 30 years ago, Meyer and coworkers reported the first ruthenium-based catalyst for water oxidation, known as the “blue dimer” (BD). This catalyst may be considered as an artificial analog of the oxygen-evolving complex (OEC) in the Photosystem II (PS II) as they both undergo oxidative activation by proton coupled electron transfer (PCET) to reach higher oxidation states where water oxidation occurs [5,6,7,8]. It is also the most studied catalyst of water oxidation reactions. Experimental data on its reaction mechanism obtained over the past 30 years are summarized in Figure 1. Water oxidation is a complicated process involving several transition states and intermediates, many of which are short-lived [6,9,10,11,12,13]. For this reaction the H/D isotope effect may help to identify reaction steps coupled to proton transfer as well as stabilize some of the highly-reactive transient intermediates in the blue dimer catalytic cycle. 

In the catalytic scheme in Figure 1, blue dimer[3,3] and [3,4] are known to be stable forms of the catalyst, numbers in square brackets denote the oxidation state of the ruthenium (Ru) centers. BD[4,5] and [5,5] intermediates are proposed to be active towards O–O bond formation with possible generation of peroxo intermediates. When catalysis is initiated by using ceric ammonium nitrate at acidic pH, BD[3,4]′ builds up in reaction mixtures and its oxidation appears to be a rate limiting step [11]. Of the blue dimer (BD) oxidation states, [4,5], [5,5] and BD[3,4]′ have been characterized to an extent previously [10,11,12,14,15] and in this work. 

The blue dimer BD[4,5] intermediate had been previously shown to be thermodynamically unstable at pH below 2 and was thus generated by electrolysis at pH 7 at a potential of 1.2 V and also by hypochlorite oxidation in a phosphate buffer [9]. Its electron paramagnetic resonance (EPR) spectrum had characteristic components at g*_xx_* = 2.039, g*_yy_* = 1.995, g*_zz_* = 1.895 [9]. In recent work, we demonstrated that the [4,5] intermediate can be detected in 0.1 M HNO_3_ at very short times after addition of the oxidant Ce(IV) [11]. Extended x-ray absorption fine structure (EXAFS) analysis as well as resonance Raman confirmed assignment of oxidation state and presence of a short Ru=O bond [11]. In addition, EPR results obtained for ^17^O labeled BD[4,5] provided further insights into its electronic structure, demonstrating high spin density on the Ru^V^=O oxygen consistent with a radicaloid intermediate [12]. The protonated state of the Ru^IV^ center in BD[4,5] in 0.1 M HNO_3_ (whether it is Ru^IV^–OH or Ru^V^=O) is not known with certainty. EXAFS fits carried out to model the protonation states Ru^V^=O, Ru^IV^=O (two short Ru=O distance per BD molecule) *versus* Ru^V^=O, Ru^IV^–OH (one short Ru=O distance in the BD molecule) have shown a better fit quality for the Ru^V^=O, Ru^IV^–OH formulation (Tables 1 and S1, Figure S1 in [12]). 

The BD[5,5] intermediate is included in Figure 1 based on a previous literature report [9] as well as a recent manuscript by Stull *et al.* [16] which summarizes conditions for observation of this intermediate in the BD catalytic cycle. The authors, however, do not explain the origin of the EPR signal assigned to BD[5,5] which has two interacting Ru *d^3^* centers and thus is not compatible with the spin S = 1/2 electronic configuration. In another report, it has been reported that a spectroscopic signature, for BD[5,5] cannot be observed due to its rapid rate of water oxidation [6]. We have preliminary data that the species reported there as BD[5,5] is actually another form of BD[4,5]. (A detailed analysis will be presented elsewhere).

The exact molecular structure of BD[3,4]′ remains unknown. Resonance Raman experiments previously detected a 683 cm^−1^ band which underwent a 46 cm^−1^ shift upon ^16^O/^18^O substitution [11]. Assignment of this band to the O–O vibration coupled to the Ru-O-Ru bridge was premature [11], as an isotope labeling experiment in 50% H_2_^16^O and 50% H_2_^18^O did not reveal the 1:2:1 intensity pattern expected for the O–O fragment (see Figure S6, [12] and [16]). This still does not rule out the possibility that the detected vibration is a Ru–O stretch in the Ru–OOH peroxide fragment. The accumulation of BD[3,4]′ under different oxidizing conditions is an interesting new topic and the properties of this intermediate will be further analyzed by Discrete Fourier Transform (DFT) computation of Raman frequencies and by X-ray scattering.

This paper describes H_2_O/D_2_O kinetic isotope effects (KIE) in water oxidation by the blue dimer. Isotope effects appear in Ce(IV) oxidation of BD[3,4] to BD[4,5] and Ce(IV) oxidation of BD[4,5] in its reaction with water with the formation of BD[3,4]′. A kinetic isotope effect (KIE) for the latter of 2.1–2.5 was determined experimentally by a combined UV-Vis stopped flow and EPR analysis. This value is consistent with observations made earlier for a single site water oxidation catalyst where it was concluded that the mechanism of O–O bond formation was Atom Proton Transfer (APT). In this mechanism, O–O bond formation occurs in concert with loss of a proton to a second water molecule or cluster [17]. By contrast, the H_2_O/D_2_O KIE for the overall reaction is small. This comparison reveals that the rate limiting step or steps in the overall water oxidation catalytic cycle is not the O–O bond forming step and is consistent with rate limiting oxidation of BD[3,4]′ by Ce(IV). 

## 2. Results and Discussion

### 2.1. Determination of the KIE (k_H2O_/k_D2O_) for Oxygen Evolution with Blue Dimer Catalyst 

**Figure 2:**
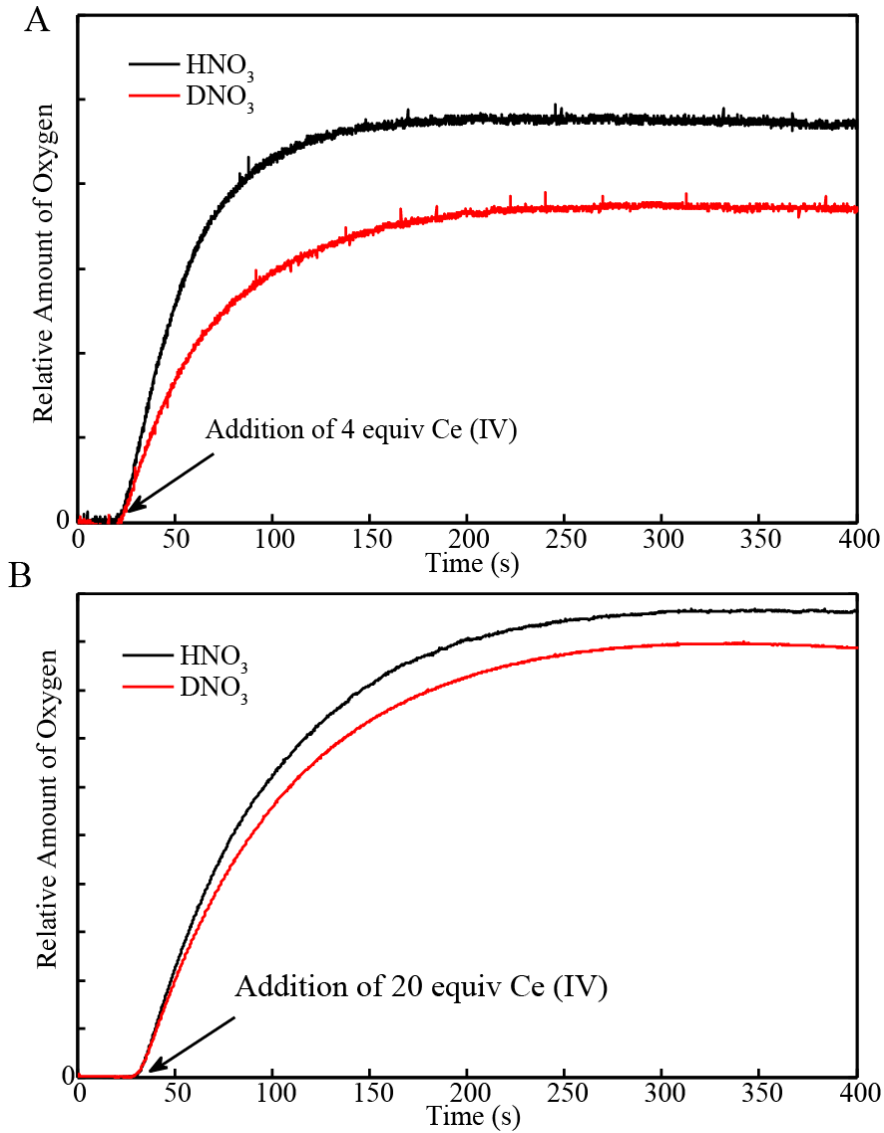
Kinetics of O_2_ evolution recorded with oxygen electrode immersed in (**A**) H_2_O and (**B**) D_2_O solution of the blue dimer[3,3] (0.1 mM in 0.1 M HNO_3_) after addition of 4 equiv of Ce(IV) to induce a single turnover as well as 20 equiv of Ce(IV).

The blue dimer catalyst evolves oxygen when Ce(IV) oxidant is added at acidic pH. Oxygen evolution measurements were carried out with an oxygen electrode immersed in the reaction mixture (0.1 M HNO_3_, See Experimental Section). Figure 2A shows the profile of the O_2_ evolution from a solution of 0.1 mM of blue dimer catalyst in 0.1 M HNO_3_. In order to induce a single turnover, 4 equiv of Ce(IV) were added to 0.1 mM BD[3,3] catalyst. O_2_ evolution continued for about 5 minutes in H_2_O and D_2_O and resulted into evolution of 0.044 μmol and 0.034 µmol of oxygen (Figure 2A, Table 1) respectively. The initial rate of oxygen evolution determined within the first 30 s was 9.5 × 10^−4^ µmol/s and 6.1 × 10^−4^ µmol/s in H_2_O and D_2_O, respectively (Figure 2A). When excess Ce(IV) (20 equivalents) was added to solutions of the BD[3,3] catalyst, oxygen evolution resulted in 0.36 μmol and 0.33 μmol of O_2_ in H_2_O and D_2_O respectively and the maximum rate of O_2_ evolution was 4.3 × 10^−3^ µmol/s and 3.8 × 10^−3^ µmol/s, respectively (Figure 2B, Table 1). The turnover frequency in H_2_O/D_2_O was determined by the ratio of the initial rate of oxygen evolution when excess Ce(IV) was added, multiplied by the total volume of catalyst used (1000 µL) over the concentration of BD[3,3] catalyst (0.1 mM). The turnover frequency (TOF) in H_2_O was determined to be 0.043/s whereas reaction in D_2_O yielded a TOF of 0.038/s. This suggests that one catalytic cycle in H_2_O and D_2_O is about 23 s and 26 s, respectively. This small difference suggests that the rate-limiting step (in this case it is proposed to be oxidation of [3,4]′, see below and in [11]) is not significantly affected. The values obtained for the turnover frequencies are comparable to the time frame for formation of the oxygen evolving BD[3,4]′ intermediate (7 and 25 s in H_2_O and D_2_O respectively, see below for details). In summary, oxygen evolution in BD is only slightly slower when the reaction is carried out in D_2_O compared to H_2_O. This arises from slower kinetics for the formation of the oxygen evolution intermediate BD[3,4]′ as will be demonstrated later in Section 2.2. 

**Table 1:** Rate of oxygen evolution and total oxygen evolution for 0.1 mM BD in H_2_O and D_2_O solution at pH = 1.

Sample	Initial Rate of Oxygen Evolution (µmol/s)	Total Oxygen Evolved (µmol)	Turnover Frequency *
BD + 4 equiv H_2_O	9.5 × 10^−4^	0.044	0.043/s
BD + 20 equiv H_2_O	4.3 × 10^−3^	0.36	
BD + 4 equiv D_2_O	6.1 × 10^−4^	0.034	0.038/s
BD + 20 equiv D_2_O	3.8 × 10^−3^	0.33	

* was calculated using initial rate of oxygen evolution.

### 2.2. Stopped-Flow Kinetic Analysis of Blue Dimer Water Oxidation Cycle 

**Figure 3:**
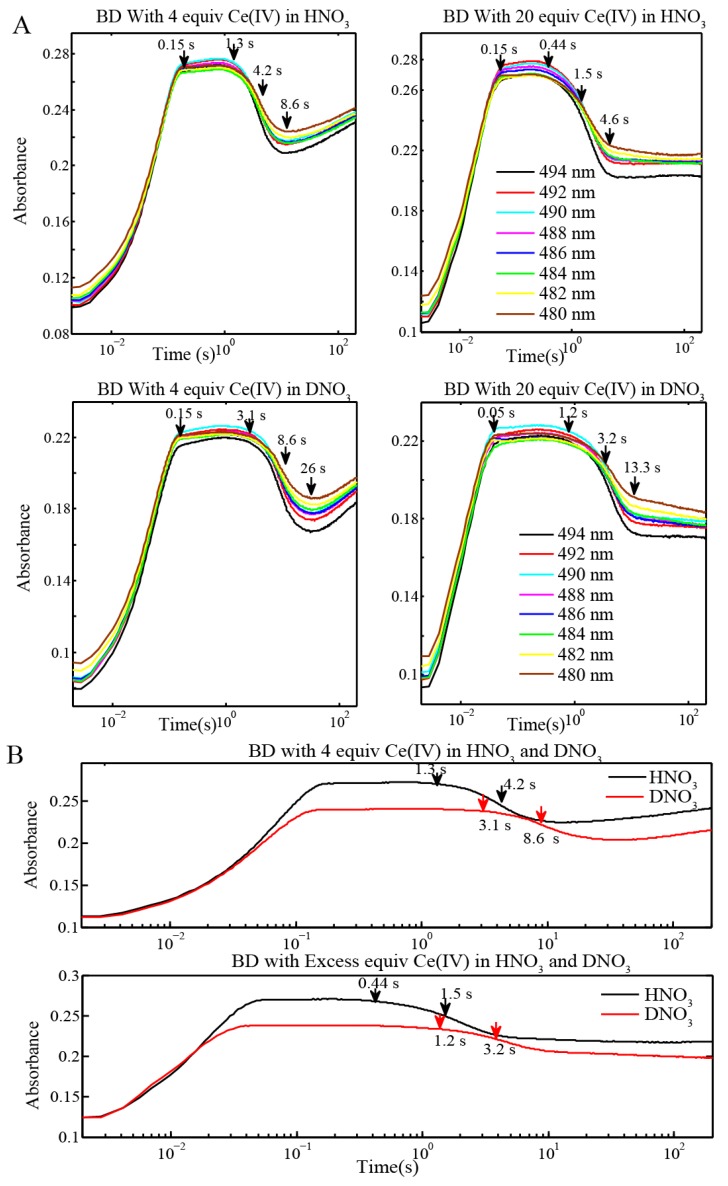
(**A**) Stopped-flow UV-Vis measurement of BD[3,3] (0.1 mM) oxidation with 4 equiv and 20 equiv of Ce(IV) at pH 1 (HNO_3_) in H_2_O and in D_2_O; (**B**) Comparison of the absorbance traces at 480 nm in H_2_O and D_2_O for BD[3,3] with 4 equiv and 20 equiv Ce(IV) at pH 1.

Stopped-flow UV-Vis kinetic analysis of the BD oxidation was carried out in order to delineate which steps of the catalytic cycle are affected by D_2_O and, thus, are responsible for slowing down catalytic water evolution. In a first set of experiments, the stable catalyst BD[3,3] at a concentration of 0.1 mM was used as starting material. UV-Vis measurements were carried out under the same stoichiometric conditions as in the oxygen evolution measurements, by adding 4 equiv and excess (20 equiv) of Ce(IV) to 0.1 mM BD[3,3] in H_2_O and D_2_O, (Figure 3A,B). The UV-Vis spectra of the stable forms of the BD[3,3] (637 nm—absorption maximum in 0.1 M HNO_3_) and [3,4] (494 nm—absorption maximum in 0.1 M HNO_3_) as well as absorbance-time changes following addition of Ce(IV) were monitored. Kinetic of BD[3,3] oxidation to [3,4] with Ce(IV) was studied earlier and a rate constant *k* ~ 2 × 10^4^ M^−1^ s^−1^ at pH 0 in HClO_4_ was obtained [6]. Similar rate constants of 1.22 × 10^4^–2 × 10^4^ M^−1^ s^−1^ were observed in this work for BD[3,3] oxidation to BD[3,4] in H_2_O and D_2_O at pH 1 (Table 2). 

**Table 2:** Rate constants derived from kinetic modeling of the reaction of 0.1 mM BD[3,3] with 4 equiv of Ce(IV) in HNO_3_ and DNO_3_ at pH = 1. UV-Vis absorbance curves and kinetics fits are shown in Figure 3A and Figure 4.

Reaction (Fits from BD[3,3] + 4 equiv Ce(IV) in H_2_O and D_2_O)	Rate Constants in H_2_O (M^−1^ s^−1^)	Rate Constants in D_2_O (M^−1^ s^−1^)
BD[3,3] + Ce(IV) = BD[3,4]	2.0 × 10^4^ ± 280	1.2 × 10^4^ ± 200
BD[3,4] + Ce(IV) = BD[4,5]	1000 ± 42	470 ± 20
BD[4,5] + Ce(IV) = BD[3,4]′	300 ± 10	120 ± 12

**Figure 4:**
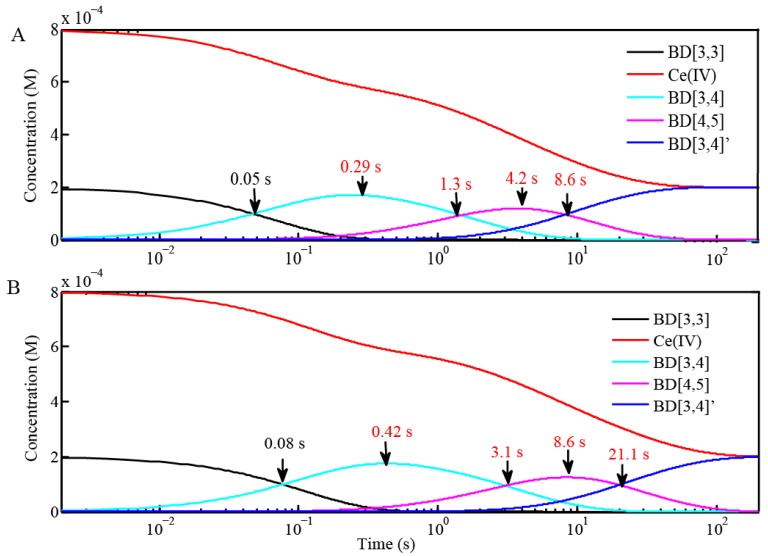
Kinetic modeling of the reaction of 0.1 mM BD[3,3] with 4 equiv of Ce(IV) at pH 1 in (**A**) H_2_O and (**B**) D_2_O. Concentration profiles of BD[3,3], BD[3,4], BD[4,5], BD[3,4]′ and Ce(IV) are shown. Rate constants are given in Table 2. UV-Vis absorbance kinetic results for these reactions are shown in Figure 3A,B.

Oxidation of BD[3,3] by addition of 4 equiv and excess Ce(IV) (20 equiv) is accompanied by a shift in the absorption maximum from 494 nm to 480 nm (Figure 3A). Note that the absorption peak width (about 100 nm) is much larger than the shift in the absorption maximum which complicates the interpretation of the UV-Vis data. The absorption maximum at 480–482 nm, by further EPR and resonance Raman analysis, corresponds to BD[4,5]. When excess Ce(IV) is added, decay of BD[3,4] is accompanied by formation of BD[4,5]. It is evident from the inflection points (Table 3) that oxidation of BD[3,4] is slower in D_2_O (Figure 3A,B). For instance, the inflection points for the UV-Vis absorption decay of BD[3,4] to form BD[4,5] were found at 4.2 s and 8.6 s, respectively, when only 4 equiv of Ce(IV) is added to BD[3,3] (Figure 3A). These values are in exact agreement with the time scale for maximum BD[4,5] formation in H_2_O and D_2_O, 4.2 and 8.6 s, respectively (Figure 4). In addition, when 4 equiv of Ce(IV) is added to BD[3,3], the times at which BD[3,4]′ starts to form and exceeds the concentration of BD[4,5] were 8.6 s and 21.1 s in H_2_O and D_2_O, respectively (Figure 4) . These times are also in agreement with the times determined for the rise of BD[3,4]′ from UV-Vis time-dependent spectral data in Figure 3A. When excess (20 equiv) Ce(IV) is added to BD[3,3] (Figure 3A,B), the inflection points for decay of BD[3,4] forming BD[4,5] were 1.5 s and 3.2 s in H_2_O and D_2_O (Table 3) displaying a similar KIE of k_H2O_/k_D2O_ of 2–2.1.

Stopped-flow UV-Vis measurements were also conducted at a higher concentration of BD (0.25 mM) with BD[3,4] as the starting form of the catalyst to simplify the catalytic cycle. These conditions parallel the sample preparation for EPR and resonance Raman analysis. The inflection points (indicated in Figure 3A,B) for the reaction of BD[4,5] with H_2_O to form BD[3,4]′ were compared at 0.1 mM and 0.25 mM catalyst concentrations (Table 3). 

**Table 3:** Inflection points from the reaction of 0.1 mM BD[3,3] + 4 equiv and 20 equiv Ce(IV) in HNO_3_ and DNO_3_ at pH = 1 and 0.2 mM BD[3,4] + 20 equiv Ce(IV) in HNO_3_.

Reactions	Inflection point for reaction of BD[4,5] in H_2_O	Inflection point for reaction of BD[4,5] in D_2_O
BD + 4 equiv Ce(IV) (0.1 mM for Oxygen Evolution) (Figure 3 and Figure 4)	4.2 s	8.6 s
BD + 20 equiv Ce(IV) (0.1 mM for Oxygen Evolution) (Figure 3)	1.5 s	3.2 s
BD + 20 equiv Ce(IV) (0.25 mM for EPR and resonance Raman) (Figure 6C)	645 ms	1.45 s

Note: UV-Vis absorbance-time traces and kinetic fits are shown in Figure 3 and Figure 4 for reaction of 0.1 mM BD[3,3] with 4 equiv and 2 equiv Ce(IV) and in Figure 6C for reaction of 0.25 mM BD[3,4] with 20 equiv Ce(IV).

Based on fitting the decay of BD[3,4] absorbance-time traces, rate constants for the formation of BD[4,5] were found to be 1000 ± 42 M^−1^ s^−1^ and 470 ± 20 M^−1^ s^−1^ in H_2_O and D_2_O, respectively (Figure 4, Table 2). Fits of the rise in absorbance, corresponding to the formation of BD[3,4]′, gave k = 300 ± 10 M^−1^ s^−1^ and 120 ± 12 M^−1^ s^−1^ in H_2_O and D_2_O, respectively (Figure 4, Table 2. Rate constant comparisons of the data in Table 2 and comparisons of inflection points and concentration mapping profiles (Table 3, Figure 3, Figure 4 and Figure 6C) give an overall KIE of k_H2O_/k_D2O_ = 2.1–2.5.

### 2.3. EPR and Resonance Raman Demonstration of Extended Lifetime of BD[4,5] in D_2_O 

**Figure A1:**
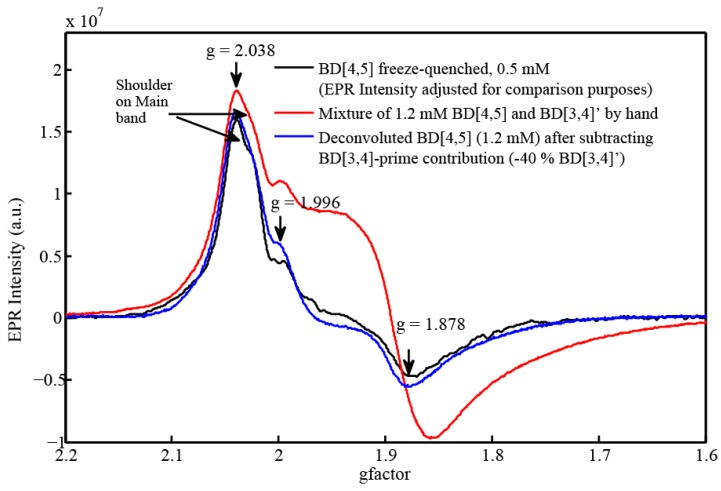
EPR spectra of BD[4,5] freeze-quenched at higher concentration (0.5 mM, pH 1 in HNO_3_) in comparison with mixture of 60% BD[4,5] and 40% BD[3,4]′ prepared by manual mixing (1.2 mM, pH 1 in HNO_3_). BD[4,5] after subtracting 40% of BD[3,4]′ contribution is also shown (1.2 mM, pH 1 in HNO_3_). The shoulder on the main band is emphasized by arrows and the g-tensors are shown. G-tensor components at 2.038 and 1.996 shown by arrows are in agreement with those reported in Figure 5B in [9]. Please note that the EPR intensity of BD[4,5] prepared by freeze quench(0.5 mM) was adjusted for comparison purposes.

The intermediate BD[4,5] has a characteristic EPR spectrum reported previously with g-tensor (g*_xx_* = 2.039, g*_yy_* = 1.995, g*_zz_* = 1.895) [9,11], later simulations gave g*_xx_* = 2.03, g*_yy_* = 1.98, g*_zz_* = 1.87 [12]. This intermediate is unstable and, thus, freeze quench techniques were used previously to prepare BD[4,5] (Figure A1) [11]. Figure A1 compares the EPR spectrum of pure BD[4,5] prepared by freeze quench (0.5 mM catalyst concentration) with a mixture of BD[4,5] and BD[3,4]′ (40%) observed in a sample prepared by manual mixing with the catalyst at 1.2 mM. A shoulder on the high field side of the g = 2.03–2.04 component is present in both samples. 

**Figure 5:**
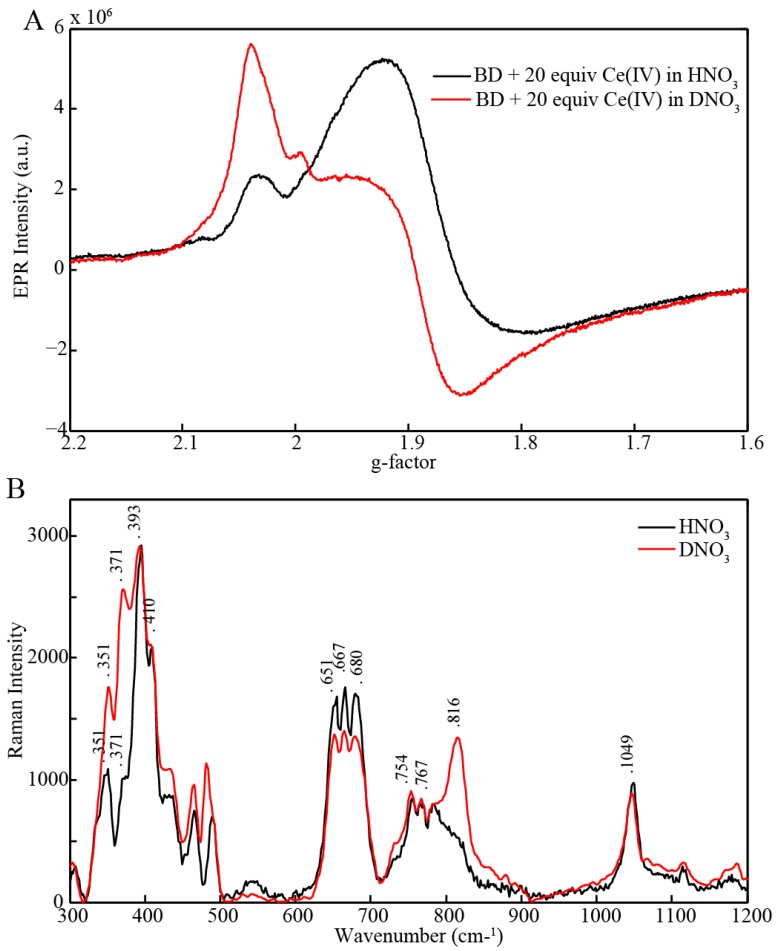
(**A**) X-Band electron paramagnetic resonance (EPR) of 1 mM BD[3,4] mixed with 20 equiv Ce(IV) by hand in HNO_3_ and DNO_3_, pH = 1; (**B**) Resonance Raman of 1 mM BD[3,4] with 20 equiv Ce(IV) in HNO_3_ and DNO_3_, pH = 1 carried out in parallel with EPR measurements.

We notice, however, that when BD[3,4] is oxidized with excess of Ce(IV) in D_2_O (pH = 1, 1 mM HNO_3_) and samples are manually frozen (approximately 30 s after mixing), the EPR spectrum is dominated by the BD[4,5] signal (Figure 5A). When the same experiment is performed in H_2_O (pH = 1, 1 mM HNO_3_) the spectrum is dominated by BD[3,4]′ (Figure 5A). These results show that BD[4,5] is stabilized in D_2_O. We attribute this to the slower rate of BD[4,5] oxidation in D_2_O compared to H_2_O. 

The same samples were analyzed by resonance Raman with excitation at 532 nm, Figure 5B. Resonance Raman measurements carried out in H_2_O (pH = 1, 1mM HNO_3_) revealed two pronounced features at 371 cm^−1^, and 395 cm^−1^ corresponding to vibrations of the Ru-O-Ru bridge in BD[4,5] and a second band at 410 cm^−1^ corresponding to the Ru–O–Ru bridge vibration in BD[3,4]′. A main band at around 683 cm^−1^ characteristic for [3,4]′ was observed. In addition, a small shoulder at 816 cm^−1^ was observed which corresponds to a Ru^V^=O vibration [9,10,12] and undergoes a 35 cm^−1^ shift to lower frequency for BD[4,5] prepared in H_2_^18^O (Figure S3, [12]). Based on earlier reports, this shift is in good agreement with assignment of the 816 cm^−1^ band to a Ru^V^=O vibration. The band at 816 cm^−1^ was clearly resolved for samples prepared in D_2_O revealing the presence of BD[4,5]. Thus, combined EPR and Raman data are consistent with stabilization of BD[4,5] in D_2_O. 

### 2.4. Time Resolved EPR Spectroscopy and Resonance Raman 

**Figure 6:**
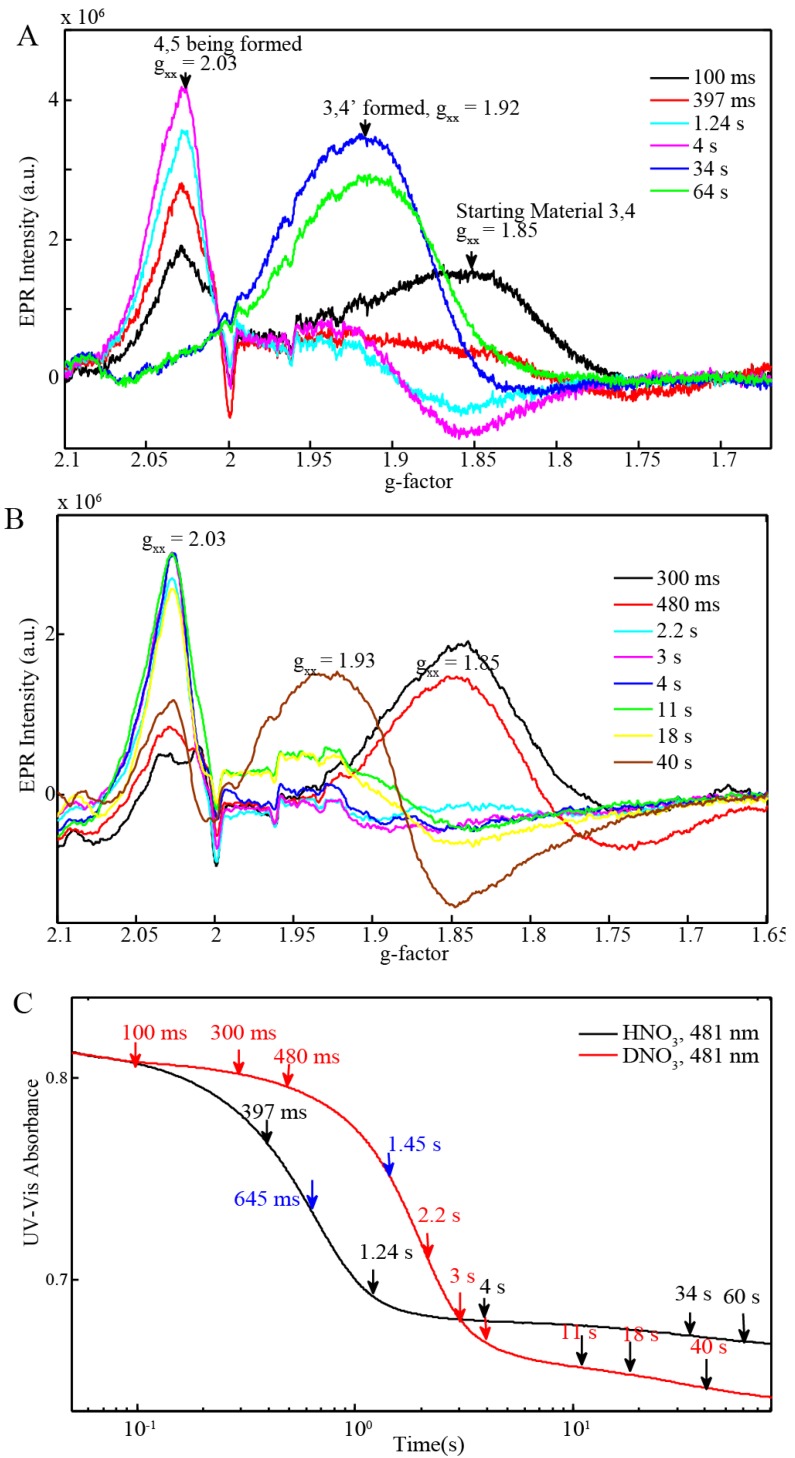
(**A**) X-Band EPR of 0.25 mM BD[3,4] with 20 equiv Ce(IV) in H_2_O freeze-quenched at different time intervals; (**B**) X-Band EPR of 0.25 mM BD[3,4] with 20 equiv Ce(IV) in D_2_O freeze-quenched at different time intervals; (**C**) UV-Vis absorbance curve at 481 nm showing times at which samples were freeze-quenched in H_2_O and D_2_O (The inflection points of both absorbance curves are shown in blue).

In order to further assess the dynamics of the oxygen evolving catalytic cycle, time resolved EPR spectroscopy and resonance Raman measurements were carried out on frozen samples prepared by stopped-flow mixing of BD[3,4] with 20 equiv excess of Ce(IV) with consecutive freeze quench cycles at defined time intervals. EPR spectra of samples prepared in 0.1 M HNO_3_ [11] and DNO_3_ are shown in Figure 6A,B. Parallel stopped-flow UV-Vis absorption kinetic measurements are shown in Figure 6C. Figure 6C shows the characteristic changes in absorbance at 481 nm upon oxidation of BD[3,4] with arrows indicating the times at which samples were collected for EPR analysis in HNO_3_ and DNO_3_. These experiments were repeated several times and similar results were obtained. Upon comparing Figure 6A,B, we see that the intensity of the BD[3,4] signal with g-factor around 1.85 quickly decreases giving rise to the transient intermediate BD[4,5] which is observed with a small contribution of BD[3,4] at 397 ms and without a contribution from BD[3,4] at 1.24 s (Figure 6A). These times agree well with the inflection point for BD[4,5] formation at 645 ms (Table 3, Figure 6C). BD[4,5] persists for ~4 s and reacts with water forming BD[3,4]′ at ~30 s. The latter evolves O_2_ and slowly forms BD[3,4]. We observe a mixture of the BD[3,4]′ and BD[3,4] at ~1 min. This reaction is slowed by a factor of ~2.1–2.5 in DNO_3_. Upon addition of excess Ce(IV) in DNO_3_, the BD[3,4] signal persists until 480 ms before forming intermediate BD[4,5]. BD[4,5] is obtained in mixtures with BD[3,4] at 480 ms. There is no BD[3,4] in the mixture at 2.2 s (Figure 6B). This agrees with the inflection point at 1.45 s observed with UV-Vis kinetic measurements for the formation of BD[4,5] in D_2_O (Figure 6C). The BD[4,5] intermediate persists for a longer time, ~11 s, before forming a mixture that contains some BD[3,4]′. A 40%–50% presence of BD[4,5] is still observed in DNO_3_ at 30 s (Figure 6B).

**Figure 7:**
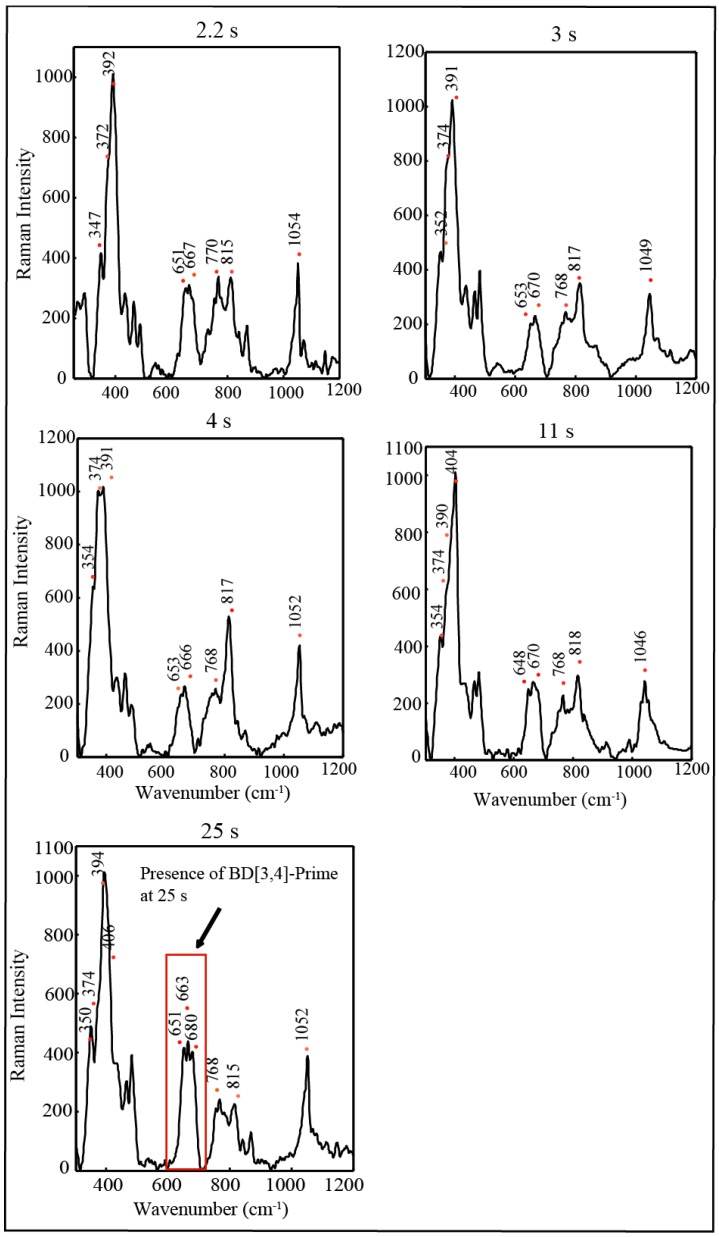
Resonance Raman measurements of 0.25 mM BD[3,4] with 20 equiv Ce(IV) in DNO_3_, pH = 1 freeze quenched at time intervals shown in Figure 6B.

**Figure A2:**
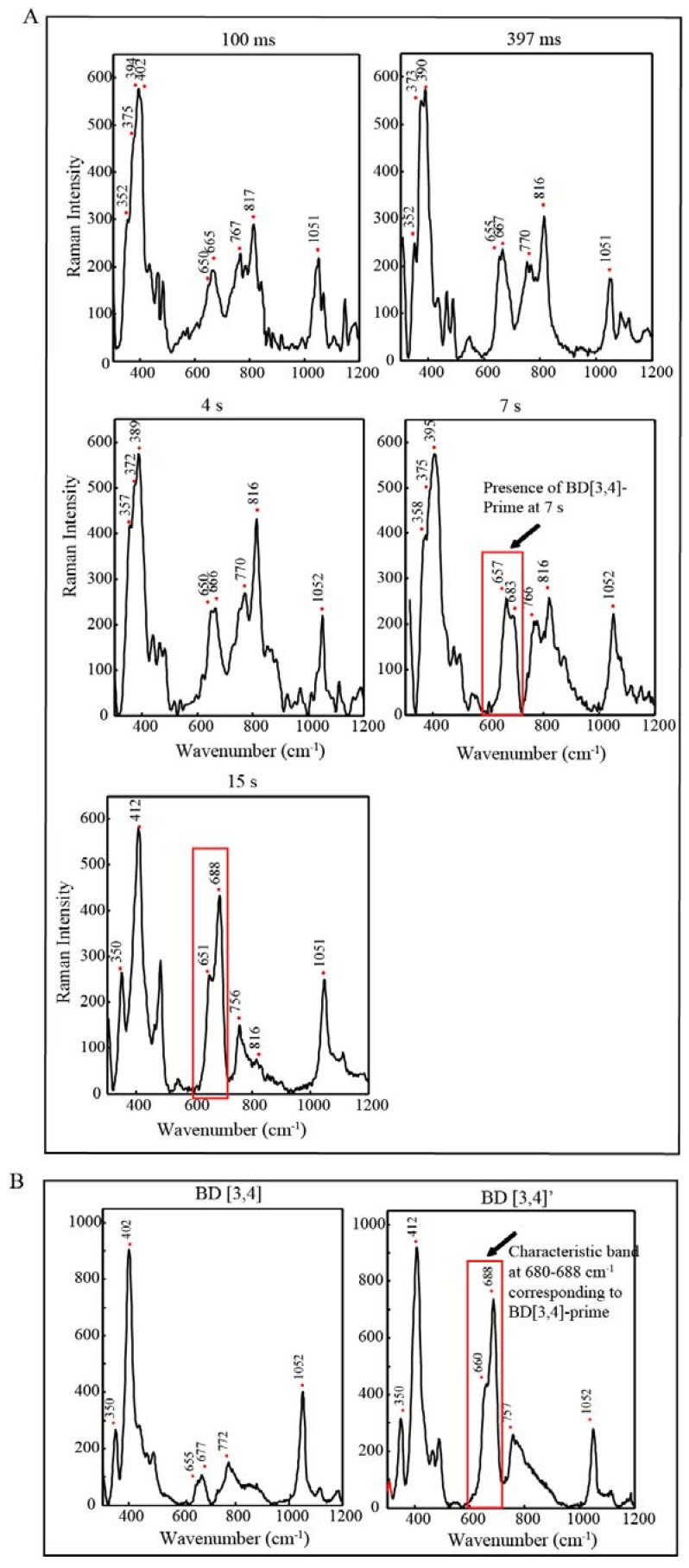
(**A**) Resonance Raman measurements of 0.25 mM BD[3,4] with 20 equiv Ce(IV) in HNO_3_, pH 1 freeze quenched at some time intervals shown in Figure 6A; (**B**) Resonance Raman of 1 mM BD[3,4] and BD[3,4]′, pH 1 in HNO_3_.

Parallel resonance Raman measurements were carried out by adding 20 equiv of Ce(IV) to BD[3,4] in HNO_3_ and DNO_3_ with the samples freeze-quenched at the same times as for the EPR measurements from 100 ms to 15 s in HNO_3_ (Figure A2) and 2.2–25 s in DNO_3_ (Figure 7). 

The 816–818 cm^−1^ peak assigned to the Ru=O stretch [10,18,19] was observed in samples prepared from 100 ms to 7s, Figure A2 in HNO_3_ with the peak present until 25 s in DNO3 (Figure 7). In HNO_3_, this peak is transient and with appearance of the 683 cm^−1^ band corresponding to BD[3,4]′ already at 7 s and predominant at 15 s (Figure A2). However in DNO_3_, the 683 cm^−1^ peak only arises at 25 s. Resonance Raman measurements on control samples of BD[3,4] and BD[3,4]′ have been characterized before [10,11] and are also shown (Figure A2).

## 3. Experimental Section 

### 3.1. Sample Preparations

Throughout this study the blue dimer was used as the PF_6_^−^ and ClO_4_^−^ salts, and no differences between the two were observed. [((bpy)_2_Ru^III^(H_2_O))_2_O](PF_6_)_4_ was prepared from [((bpy)_2_Ru^III^(H_2_O))_2_O](ClO_4_)_4_, as previously described [5] via salt metathesis by adding NH_4_PF_6_ to an aqueous solution of the ClO_4_^−^ salt. The blue dimer cation was purified by chromatography on LH-20 Sephadex. Blue dimer [3,4] was prepared by oxidation with one equivalent of ceric ammonium nitrate, Ce^IV^(NH_4_)_2_(NO_3_)_6_·4H_2_O. It was used as it is or after additional purification and re-crystallization with no differences noticed. Purity of blue dimer[3,3] and [3,4] was verified by comparison with known electrochemical and electronic spectra [5]. Ultrapure (Type 1) water (resistivity 18.2 MΩ cm at 25 °C, TOC 4 μg/L) sourced from a Q-POD unit of Milli-Q integral water purification system (Millipore) was used for solutions. All samples were prepared in 0.1 M HNO_3_ acid or 0.1 M DNO_3_ acid, pH 1.0 (Catalog No. 225711 and 151882, respectively from Sigma Aldrich). Oxidant solutions were prepared fresh daily by dissolving Ce (NH_4_)_2_(NO_3_)_6_·4H_2_O in 0.1 M HNO_3_, 0.1 M DNO_3_ and 0.1 M CF_3_SO_3_H.

### 3.2. Oxygen (O_2_) Evolution Measurements

Oxygen evolution was measured with a PC operated Clark type polarographic oxygen electrode from Oxygraph System (Hansatech Instruments Ltd.). The sample was housed within a hermetic borosilicate glass reaction vessel thus preventing penetration of any atmospheric oxygen. Calibration was carried out by measurements of the signal from O_2_-saturated water in an open reaction vessel. Sodium dithionite, an oxygen-depleting agent, was added to the water and the drop in the signal was related to the solubility of oxygen in water at room temperature (262 µmol/L). The glass vessel was thoroughly washed with water and 1 mL of 0.1 mM blue dimer[3,3] was added. A defined number of Ce(IV) equivalents were carefully added by means of a Hamilton syringe into the chamber through a septum cap and oxygen evolution was measured as a function of time. 

### 3.3. UV-Vis, Stopped Flow, Freeze Quench

SX20 Stopped-Flow UV-Vis Spectrometer (Applied Photophysics Ltd.) with a dead time of 0.5 ms was used to follow the reactions of blue dimer [3,4] with consecutive equiv of Ce(IV). Changes in the absorbance were monitored from times as early as 1 ms after Ce(IV) addition. Cuvettes with path lengths of 2 mm and 10 mm were utilized to study changes in the absorbance of concentrated 0.25 × 10^−3^–0.1 × 10^−3^ M and less concentrated samples 0.5 × 10^−4^–1 × 10^−4^ M, respectively. No principal differences were observed when blue dimer was oxidized in 10^−4^ M *versus* 10^−5^ M concentration ranges. Increases in the blue dimer concentration only resulted in increases in reaction rates as expected from the rate law. 

UV-Vis absorption measurements were conducted in parallel with other spectroscopic techniques, namely EPR and resonance Raman. In order to measure samples using EPR and resonance Raman, fast freeze-quenching of reaction mixtures was performed by using an SFM 20 Stopped–Flow System (Bio-Logic Science Instruments). The apparatus is equipped with an umbilical connector with a built-in ejection nozzle at the end of the ageing loop which sprays the aged reaction mixtures into pre-cooled liquid pentane at −120 °C. This setup allows for freezing of reaction mixtures starting 1 ms after reagent mixing. Warning: Liquid pentane is flammable. Great care should be taken when storing and handling. Samples were collected from liquid pentane with EPR collection kits. To ensure that intermediates do not react with pentane at −120 °C, samples were also collected by spraying reaction mixtures into liquid nitrogen. The blue dimer catalytic cycle was observed using pentane (−120 °C) as well as liquid nitrogen as cryogens. Liquid nitrogen provides a slower freezing rate and should not be used for monitoring short (less than 2 s) reactions.

### 3.4. EPR Measurements

Low-temperature X-band EPR spectra were recorded by using a Bruker EMX X-band spectrometer equipped with a X-Band CW microwave bridge. The sample temperature was maintained at 20 K, unless otherwise indicated, by use of an Air Products LTR liquid helium cryostat. Spectrometer conditions were as follows: microwave frequency, 9.65 GHz; field modulation amplitude, 10 G at 100 kHz; microwave power, 31.70 mW. Standard EPR sample tubes were filled with sample through all of the resonator space and whenever relative signal intensities are discussed, measurements were conducted on the same day in the same conditions to allow direct comparison of the signal intensities. Field calibration was checked *versus* a DPPH standard. 

### 3.5. Resonance Raman Measurements

Low-temperature resonance Raman spectra were recorded using an XploRa Horiba Raman microscope at 532 nm excitation. The samples were measured on a Linkam cryostage (100 K) connected to the microscope stage below the laser beam aperture. The sample and window space of the cryostage were continuously purged with nitrogen gas to avoid frost formation and enable easy focusing on the sample. Freeze quenched samples are more prone to damage and were measured with 2.5 mW excitation power. Samples prepared by hand were measured with 10 mW laser excitation power. Scans were recorded with shortest 2 s exposure and no laser induced damage was observed in at least 10 scans for freeze quenched samples and 100 scans for hand-prepared samples.

## 4. Conclusions 

Isotope effects on the kinetics of intermediate formation in the catalytic cycle for water oxidation by the blue dimer have been analyzed by a combination of UV-Vis stopped flow kinetics, oxygen evolution, EPR spectroscopy and resonance Raman measurements. Stopped flow kinetics coupled with parallel EPR and Raman measurements were used to delineate the steps in the overall catalytic cycle. The rate of Ce(IV) oxidation of BD[4,5] with subsequent reaction with water and formation of BD[3,4]′ was decreased by a factor of 2.1–2.5 in D_2_O consistent with Atom Proton Transfer. The overall oxygen evolution rate was affected to a far lesser degree showing that the rate-limiting step in the overall mechanism is *not* water oxidation consistent with the rate-limiting oxidation of BD[3,4]′.

Time resolved EPR and Raman measurements have shown that BD[4,5], with a characteristic EPR spectrum and Raman vibration for Ru=O at 816 cm^−1^, persists for longer times in D_2_O than in H_2_O. Our results add to the literature utilizing flow kinetic methods and deuterium isotope effects to resolve individual steps in catalytic mechanisms with examples in dehydrogenase and oxygenases over CeO_2_ supported Pt-Co catalysts, as well as, cytochrome P450-catalyzed reactions [20,21,22].
